# Determining the Usefulness of Selected Laboratory Markers of Inflammation in Qualifying Patients for T2 Biosystems Determination

**DOI:** 10.1002/jcla.70021

**Published:** 2025-03-27

**Authors:** Mateusz Szymański, Małgorzata M. Skiba, Małgorzata Piasecka

**Affiliations:** ^1^ Human Anatomy Department Medical University Lublin Poland; ^2^ Intensive Care Unit Stefan Cardinal Wyszyński District Specialist Hospital Lublin Poland

**Keywords:** ESKAPE, modern microbial diagnostics, T2 bacteria, T2‐biosystems, T2Dx

## Abstract

**Background:**

Improving treatment outcomes sepsis requires early recognition, the implementation of appropriate treatment, and targeted antimicrobial therapy. Nowadays, microbiological diagnostic methods are available to accelerate microbiological diagnosis, thereby reducing the time needed to implement targeted antibiotic therapy. One method for rapid diagnosis is the amplified magnetic resonance imaging—T2 Biosystems, USA (T2Dx). This method enables the identification of pathogens directly from a blood sample (approximately 4 mL) within about 3.5 h. The use of the “T2 Resistance” panel additionally allows for the detection of the most common bacterial resistance mechanisms in about 4–5 h. The disadvantage of the T2Dx method is the limited number of microorganisms it can detect. The objective of the study was to evaluate the effectiveness of using selected inflammatory parameters to accurately qualify patients (positive result) for T2Dx testing.

**Methods:**

We have made a retrospective evaluation of selected inflammatory parameters in order to determine which parameters are the best indicators for good qualification of patients.

**Results and Conclusion:**

A single analysis of parameters such as C‐reactive protein (CRP), white blood cells (WBC), #neutr, #lymph, and neutrophil‐to‐lymphocyte ratio (NLR) is not a good indicator that could be used as an additional tool facilitating patient qualification for T2Dx testing. The most sensitive parameter distinguishing between patients with a positive T2Dx result and those with a negative result is the measurement of IL‐6 and PCT. Proper patient qualification for T2Dx testing can significantly contribute to reducing the time to initiate targeted antibiotic therapy and may impact reducing mortality and improving long‐term treatment outcomes.

## Graphical abstract

Improving treatment outcomes sepsis requires early recognition, the implementation of appropriate treatment, and targeted antimicrobial therapy. One method for rapid diagnosis is the amplified magnetic resonance imaging—T2 Biosystems, USA (T2Dx). Proper patient qualification for T2Dx testing can significantly contribute to reducing the time to initiate targeted antibiotic therapy and may impact reducing mortality and improving long‐term treatment outcomes.

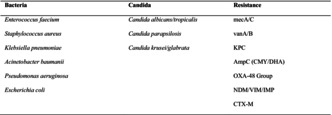

## 1 Introduction

Despite the continuous and dynamic development of many branches of medicine, sepsis and septic shock remain significant challenges for all health care systems. They are still associated with a high mortality rate, one reason for which is late diagnosis [1]. Sepsis is defined as life‐threatening organ dysfunction in response to infection. It is most often associated with the presence of bacteria in the blood, or bacteremia. Septic shock is an immediate life‐threatening condition and is characterized by the need for catecholamines despite adequate fluid therapy and elevated blood lactate levels [2]. The mortality rate in septic shock is high, reaching up to 40% [3], and the long‐term mortality rate can be as high as 56% [4]. Every hour of delay in implementing appropriate treatment for septic shock, including targeted antibiotic therapy against the potential pathogen, worsens the prognosis by several percentage points. The treatment of sepsis and septic shock is not only a challenge for the treatment team but also a serious economic burden. Sepsis is a problem for both patients with community‐acquired infections and more importantly, those already hospitalized. Improving treatment outcomes requires early recognition of sepsis, the implementation of appropriate treatment, and targeted antimicrobial therapy [1, 3, 5]. Nowadays, microbiological diagnostic methods are available to accelerate microbiological diagnosis, thereby reducing the time needed to implement targeted antibiotic therapy [6, 7, 8]. This approach not only improves treatment outcomes by reducing complications and death rates but also reduces the risk of increasing drug resistance by avoiding the use of broad‐spectrum empiric therapy [9, 10, 11, 12, 13]. One method for rapid diagnosis is the amplified magnetic resonance imaging—*T2 Biosystems*, USA (T2Dx). This method enables the identification of pathogens directly from a blood sample (approximately 4 mL) within about 3.5 h. The use of the “*T2 Resistance*” panel additionally allows for the detection of the most common bacterial resistance mechanisms in about 4–5 h. Another available panel, the *Candida* panel, enables the detection of fungi in the blood sample [14]. The disadvantage of the T2Dx method is the limited number of microorganisms it can detect. The pathogens and resistance mechanisms identified by the T2Dx method are presented in Table 1. There is still limited information in the available literature regarding the use of this technology in everyday clinical practice.

**TABLE 1:** Bacteria, fungi, and resistance mechanisms detected by T2Dx.

Bacteria	Candida	Resistance
*Enterococcus faecium* *Staphylococcus aureus* *Klebsiella pneumoniae* *Acinetobacter baumanii* *Pseudomonas aeruginosa* *Escherichia coli*	*Candida albicans/tropicalis* *Candida parapsilosis* *Candida krusei/glabrata*	mecA/C vanA/B KPC AmpC (CMY/DHA) OXA‐48 group NDM/VIM/IMP CTX‐M

C‐reactive protein (CRP) is produced by the liver in response to inflammatory processes and cell damage. It is a highly nonspecific parameter, and its value can be affected by various factors such as age, medications, physical exertion, trauma, etc. CRP has also been used as a predictive marker for cardiovascular incidents. Elevated CRP levels are also found in cancer, autoimmune diseases, and neurodegenerative diseases such as Alzheimer's disease [15, 16, 17].

Procalcitonin (PCT) is a precursor of calcitonin and, under physiological conditions, is responsible for lowering serum calcium levels. Procalcitonin is also a marker of infections caused by bacteria, fungi, and parasites. The concentration of procalcitonin increases within 2–4 h during bacterial infections and reaches its maximum level after 12–24 h. Elimination of the factor inducing the inflammatory response results in a decrease in PCT levels. Due to its half‐life of 24–35 h, procalcitonin is used to assess the severity of infection [18, 19, 20, 21].

Interleukin 6 (IL‐6) is a pleiotropic inflammatory cytokine that triggers gene expression signaling via the IL‐6R membrane receptor through the gp130 co‐receptor, a glycoprotein with a mass of 130 kDa. Gene expression is induced in this way. This pathway regulates the body's defense processes. IL‐6 also affects cellular processes, including angiogenesis, cell proliferation, and differentiation [22, 23, 24].

Leukocytes (white blood cells (WBC)) are morphotic elements of the blood. Their primary role is the body's defense function, which is mainly carried out through phagocytosis—destroying and engulfing microorganisms, degranulation, antibody production, and free radical production. Neutrophils (NEUTR), or neutrophilic granulocytes and lymphocytes (LYMPH) are types of leukocytes. The function of neutrophils is mainly phagocytosis of microorganisms. Their average life span is 2–4 days. Their activity also results in the formation of pus. Lymphocytes, which are divided into two main groups: B lymphocytes and T lymphocytes, actively participate in the body's immune response and play a significant role in destroying virus‐infected cells [25, 26, 27].

The neutrophil‐to‐lymphocyte ratio (NLR) is the quotient of neutrophils (neutrophilic granulocytes) and lymphocytes. Clinically, it is used as an indirect indicator of inflammation. Infections, congestive heart failure, gastrointestinal bleeding, or trauma can increase NLR levels. NLR is also associated with a number of civilization diseases with a confirmed inflammatory basis in their etiopathogenesis, such as obesity, diabetes, and metabolic syndrome [25, 28].

## 2 Material and Methods

The study is retrospective in nature. A total of 72 T2Dx assays conducted using the *Bacteria Panel* were analyzed. Technically defective assays or those canceled due to pre‐analytical error (7 assays) were excluded from the assessment. The final statistical analysis included 54 T2Dx assay results, performed on patients aged 26–83 years, who were hospitalized in a regional multi‐specialty hospital (Table 2).

**TABLE 2:** Patient demographics.

	*n*	*M*	Me	Min.	Max.	SD	Women	Men
Total	55	62.02	65	26	83	13.98	28	27
Group A	19	56.42	57	26	74	13.50	9	10
Group B	36	65.08	67	28	83	13.27	19	17

Abbreviations: *M*, mean age; Max, maximal age; Me, median age; Min, minimum age; *n*, numer of patents; SD, standard deviation.

All eligible patients were suspected of having sepsis or septic shock based on clinical presentation and additional tests. Among the patients included in the study were those with comorbidities such as heart failure, diabetes, chronic obstructive pulmonary disease (COPD), chronic kidney disease, and coronary artery disease. The study covered the period from October 1, 2023, to June 30, 2024. Patients were divided into two groups. *Group A* included patients who were T2Dx negative (*Target Not Detected*) and had no bacterial growth in concurrently taken blood cultures. *Group B* included patients with a positive T2Dx result. Due to the risk of false‐negative results, patients in *Group A* whose T2Dx test was negative but whose blood cultures grew bacteria not included in the *Bacteria Panel* were excluded from the study. To determine the criteria for patient qualification, laboratory results taken at the same time as the T2Dx assay were evaluated: C‐reactive protein (CRP), procalcitonin (PCT), interleukin 6 (IL‐6), and complete blood count parameters including white blood cell count (WBC), absolute neutrophil count (#neutr), absolute lymphocyte count (#lymph), and the NLR. If the laboratory tests for these parameters were performed the day before, those results were also analyzed to determine parameter trends (increase/decrease). The collected data were compiled into an Excel table and statistically analyzed using Statistica 13 (StatSoft Inc.). For quantitative features, the following were calculated: minimum and maximum values (min., max.), arithmetic mean (*M*), standard deviation (SD), and median (Me). The distribution of the analyzed features was assessed using the Shapiro–Wilk test. For quantitative features, differences between the groups were checked using Pearson's correlation test for normally distributed parameters or Spearman's correlation coefficient for other parameters. *p*‐values were calculated using Student's *t‐*test. A 5% error risk was adopted, so differences were considered statistically significant for *p* < 0.05.

## 3 Results

Group A, comprised of patients with negative T2Dx results, included 19 assays (34.55%), while Group B, which involved patients who were T2Dx‐positive, consisted of 36 patients (65.45%). In each patient who underwent T2Dx testing, blood was drawn at the same time for laboratory testing. The following inflammatory parameters were assessed: CRP, PCT, IL‐6, WBC, #neutr, #lymph, and NLR. The obtained results are shown in Table 3. The statistical significance (*p*‐value) of each parameter was evaluated. No statistically significant differences were found for CRP, PCT, WBC, #neutrophils, #lymphocytes, and NLR. A statistically significant higher concentration of IL‐6 was observed in *Group B* relative to *Group A* (Table 4). The average concentration of IL‐6 in Group A was 629.66 pg/mL, while in Group B the value reached more than double, that is12,713.42 pg/mL.

**TABLE 3:** Statistical summary of evaluated parameters for Group A and Group B.

	*n*	*M*	Me	Min.	Max.	Lower quartile	Upper quartile	SD
Group A								
CRP (mg/L)	19	212.02	212.00	60.00	421.00	142.00	282.00	101.46
PCT (ng/mL)	19	17.56	9.77	0.67	49.60	1.79	41.90	18.65
IL‐6 (pg/mL)	18	629.66	289.50	28.30	3803.00	138.00	636.00	931.43
WBC (thousands/μL)	19	17.59	15.65	7.08	30.06	12.29	25.50	7.11
#NEUTR (thousands/μL)	19	15.20	13.73	5.91	24.60	10.36	22.09	6.23
#LYMPH (thousands/μL)	19	1.17	1.02	0.35	2.63	0.70	1.53	0.60
NLR	16	16.12	14.99	5.96	30.42	8.79	20.36	7.60
Group B								
CRP (mg/L)	36	245.06	278.00	28.30	512.00	111.00	342.00	138.12
PCT (ng/mL)	36	32.82	16.02	0.17	189.50	5.16	46.80	42.03
IL‐6 (pg/mL)	35	12,713,42	2509,00	26.70	68,838,00	288.00	16,889,00	18,541,52
WBC (thousands/μL)	35	13.62	11.30	1.56	39.90	7.46	19.80	8.39
#NEUTR (thousands/μL)	34	12.16	10.13	1.39	37.47	6.84	18.47	7.70
#LYMPH (thousands/μL)	34	1.03	0.79	0.05	3.04	0.57	1.24	0.74
NLR	34	16.94	13.57	3.02	71.04	7.34	21.27	14.03

Abbreviations: #LYMPH, absolute value of lymphocytes; #NEUTR, absolute value neutrophils; CRP, C‐reactive protein; IL6, interleukin 6; *M*, mean results; Max, maximal result; Me, median results; Min, minimum result; *n*, number of results; NLR, neutrophil‐to‐lymphocyte ratioPCT, procalcitonin; SD, standard deviation; WBC, white blood cells.

**TABLE 4:** Statistically significant changes in inflammatory parameter results in both groups.

	*M* group A	*M* group B	*p*	*n* = group A	*n* = group B	SD *group A*	SD *group B*
CRP (mg/L)	245.06	212.02	0.362522	36	19	138.1229	101.46
PCT (ng/mL)	32.82	17.56	0.139471	36	19	42.03480	18.6
IL‐6 (pg/mL)	**12,713,42**	**629.66**	**0.008222**	**35**	**18**	**18,541,52**	**931.43**
WBC (thousands/μL)	13.62	17.59	0.085644	35	19	8.389366	7.11
#NEUTR (thousands/μL)	12.16	15.20	0.148173	34	19	7.704976	6.23
#LYMPH (thousands/μL)	1.03	1.17	0.479337	34	19	0.74	0.60
NLR	16.94	16.12	0.827841	34	16	14.03	7.60

*Note:* Stastistically significant changes were observed only for Il‐6.

Abbreviations: #LYMPH, absolute value of lymphocytes; #NEUTR, absolute value neutrophils; CRP, C‐reactive protein; IL6, interleukin 6; *M*, mean results; *n*, numer of results; NLR, neutrophil‐to‐lymphocyte ratio; *p*, *p*‐value; PCT, procalcitonine; SD, standard deviation, WBC, white blood cells.

On the basis of laboratory results obtained serially (2×), For this purpose, the obtained parameter result was compared with the previous result, provided that it was measured no more than 24 h earlier. In *Group A*, there were no statistically significant changes in the parameters assessed. In *Group B*, there was a statistically significant higher concentration of IL‐6 at the time of the patient's qualification for the study compared to the result recorded earlier. The average increase in the concentration of this parameter was 7.55‐fold (*p* < 0.05). A similar trend was observed for PCT. The average serum concentration of PCT increased 2.43‐fold (*p* < 0.05). There were no statistically significant differences in the dynamics of the other parameters (*p* > 0.05) (Table 5).

**TABLE 5:** Dynamics of assessed inflammatory parameters in Group B. erl.–previous measurement.

	*n*	*M*	*p*	Me	Min.	Max.	SD
CRP(mg/L)	36	245.06	0.398567	278.00	28.30	512.00	138.12
erl. CRP(mg/L)	29	215.09		178.00	37.20	480.0000	145.19
PCT (ng/mL)	**36**	**32.82**	**0.032886**	**16.02**	**0.17**	**189.50**	**42.03**
erl. PCT (ng/mL)	**26**	**13.51**		**5.43**	**0.23**	**73.700**	**18.91**
IL‐6 (pg/mL)	**35**	**12,713,42**	**0.026825**	**2509,00**	**26.70**	**68,838,00**	**18,541,52**
Erl. IL‐6 (pg/mL)	**15**	**1683,11**		**752.00**	**59.70**	**6 428,000**	**2140,29**
WBC (thousands/μL)	35	13.62	0.915582	11.30	1.56	39.90	8.39
erl. WBC (thousands/μL)	25	13.34		10.70	0.87	56.50	11.88
#NEUTR (thousands/μL).	34	12.16	0.604025	10.13	1.39	37.47	7.70
erl. #NEUTR (thousands/μL).	19	11.02		9.24	0.79	31.37	7.57
#LYMPH (thousands/μL).	34	1.03	0.236768	0.79	0.05	3.04	0.74
erl. #LYMPH (thousands/μL).	19	0.80		0.70	0.04	1.73	0.50
NLR	34	16.94	0.942802	13.57	3.02	71.04	14.03
erl. NLR	19	17.22		11.96	4.84	52.93	13.30

*Note:* The statistically significant average increase in the concentration was observed only for PCT and Il‐6.

Abbreviations: #LYMPH, absolute value of lymphocytes; #NEUTR, absolute value neutrophils; CRP, C‐reactive protein; erl, previous measurement; IL6, interleukin 6; *M*, mean results; Max, maximal result; Me, median results; Min, minimum result; *n*, numer of results; NLR, neutrophil‐to‐lymphocyte ratio; *p*, *p*‐value; PCT, procalcitonine; SD, standard deviation; WBC, white blood cells.

The correlation between the analyzed parameters in *Group A* and *Group B* was evaluated (Table 6).

**TABLE 6:** In *Group A*, Pearson correlation analysis was performed for parameters with a normal distribution (CRP, WBC, #NEUTR, NLR), and Spearman's test was used for the remaining parameters (PCT, IL‐6, WBC, #LYMPH). In *Group B*, Pearson correlation analysis was conducted for parameters with a normal distribution (CRP) and Spearman's test for the other parameters (PCT, IL‐6, WBC, #NEUTR, #LYMPH, NLR).

	*M*	SD	CRP (mg/L)	PCT (ng/mL)	IL‐6 (pg/mL)	WBC (thousands/μL)	#NEUTR (thousands/μL)	#LYMPH (thousands/μL)	NLR
Group A									
CRP (mg/L)	208.9563	110.4362	1.000000	0.355342	0.400532	0.065578	0.148030	−0.259532	0.421433
PCT (ng/mL)	16.5288	18.7803	**0.471930**	1.000000	0.461300	0.100000	0.154386	−0.163445	0.373529
IL‐6 (pg/mL)	669.7375	983.7627	0.395253	0.461300	1.000000	−0.159959	−0.079463	−0.215798	0.264706
WBC (thousands/μL)	19.1288	6.6206	0.059649	0.100000	−0.159959	1.000000	**0.959649**	**0.530757**	0.200000
#NEUTR (thousands/μL)	16.5825	5.7425	0.148030	0.436273	−0.182748	**0.976253**	1.000000	0.349710	0.399449
#LYMPH (thousands/μL)	1.2225	0.6414	−0.224956	−0.163445	−0.215798	**0.530757**	0.362039	1.000000	**−0.699044**
NLR	16.1188	7.6019	0.421433	0.322732	0.268779	0.237992	0.399449	**−0.683157**	1.000000
Group B									
CRP (mg/L)	237.22	136.09	1.000000	−0.058984	−0.208768	−0.252872	−0.238076	−0.158207	−0.013268
PCT (ng/ml)	34.86	43.31	0.163856	1.000000	**0.460429**	0.097766	0.098243	−0.236436	0.291367
IL‐6 (pg/mL)	11568.38	18478.30	0.125376	**0.460429**	1.000000	−0.275978	−0.210245	−0.244253	0.124008
WBC (thousands/μL)	14.13	8.35	−0.200939	0.097766	−0.275978	1.000000	**0.993506**	**0.498892**	0.328979
#NEUTR (thousands/μL)	12.37	7.73	−0.239896	0.098243	−0.210245	**0.993506**	1.000000	**0.452545**	**0.383040**
#LYMPH (thousands/μL)	1.04	0.75	−0.289492	−0.236436	−0.244253	**0.498892**	**0.452545**	1.000000	**−0.595904**
NLR	17.24	14.13	0.128046	0.291367	0.124008	0.328979	**0.383040**	**−0.595904**	1.000000

*Note:* The bold values mark a positive or negative correlation between biochemical parameters.

Abbreviations: #LYMPH, absolute value of lymphocytes; #NEUTR, absolute value neutrophils; CRP, C‐reactive protein; IL6, interleukin 6; *M*, mean results; NLR, neutrophil‐to‐lymphocyte ratio; PCT, procalcitonin; SD, standard deviation; WBC, white blood cells.

In *Group A*, there was a positive correlation between biochemical parameters: CRP and procalcitonin. No correlation (positive or negative) was found between biochemical parameters and blood morphology parameters. However, a positive correlation was found between the WBC and the absolute neutrophil count as well as between the WBC and the absolute lymphocyte count. A negative correlation was observed between the NLR and the absolute lymphocyte count.

In *Group B*, a positive correlation was found between the biochemical parameters: procalcitonin and interleukin‐6. Among the evaluated blood count parameters, positive correlations were found betweenleukocyte count and absolute neutrophils count, leukocyte count and absolute lymphocytes count, as well as leukocyte count and the lymphocyte‐to‐neutrophil ratio. A negative correlation was noted between the lymphocyte‐to‐neutrophil ratio and the absolute lymphocyte count.

In *Group A*, a positive correlation was observed between the levels of CRP and PCT, which was not seen in *Group B*. The similar dynamic increase in both parameters was associated with a statistically lower probability of obtaining a positive result with T2Dx. Compared to Group A, Group B exhibited three additional positive correlations among the parameters that were not present in Group A. These are the following: #NEUTR to NLR, #LYMPH to #NEUTR, and IL‐6 to PCT. With these positive correlations, there is a statistically greater chance of a positive T2Dx result.

## 4 Discussion

The rapid development of modern microbiological diagnostic methods creates favorable conditions for faster diagnosis of sepsis and septic shock. Despite increasingly better access to advanced technologies, it is neither feasible nor justified to routinely perform all available tests on every patient. Optimization of diagnostics should primarily focus on identifying high‐risk patients and expanding and/or accelerating microbiological diagnostics in such situations. Unfortunately, commonly used inflammatory markers are not sufficiently pathognomonic for sepsis and septic shock to be confidently interpreted. Therefore, simultaneous evaluation of multiple commonly used parameters, their dynamics over time, and their mutual correlations proves more effective. Additionally, it should be noted that assessing or observing a single parameter may be insufficient due to limitations associated with its interpretation. These limitations include, among others, the time of marker appearance in the bloodstream, its half‐life, normalization time, and other factors triggering parameter fluctuations, including medication use (e.g., steroids) and continuous or intermittent renal replacement therapy techniques. Correlating values of several parameters and jointly assessing their dynamics may enhance the sensitivity and specificity of interpretation. It is important to note that changes in individual parameters as well as their absence are equally significant. The study demonstrated a stronger correlation between several parameters in the group of patients with a positive T2Dx result compared to those with a negative result.

The NLR is a simple and cost‐effective marker that can be utilized as an additional tool in the diagnosis of sepsis. The necessity for further studies evaluating the utility of NLR in the diagnosis and monitoring of sepsis and septic shock is emphasized by other authors. They also report a positive correlation between NLR and prognosis as well as the occurrence of complications [28, 29]. Literature supports the prognostic value of NLR over separate evaluations of lymphocytes and neutrophils in cardiovascular diseases [30]. The results of our study in a group of patients with sepsis confirmed by the T2Dx method are consistent with these observations.

The correlation of additional test results with the clinical picture forms the basis of the correctness of the diagnostic and therapeutic process. Reports indicate the superiority of using the T2Dx system over blood cultures in terms of sensitivity [31, 32, 33]. From a practical point of view, it is impossible to perform the T2Dx assay on every patient; thus, efforts are necessary to determine the objective indications of qualification for this test. It should be noted that the application of the T2Dx system can serve as an additional helpful tool to facilitate the reduction of antibiotic therapy, and thus can favorably influence antibiotic policy [34, 35]. The T2Dx system has a high negative predictive value [33, 36].

Still, the most commonly encountered pathogens worldwide isolated from positive blood cultures are the *ESKAPE* bacteria group: *Enterococcus faecium, Staphylococcus aureus, Klebsiella pneumoniae, Acinetobacter baumannii, Pseudomonas aeruginosa, Escherichia coli* [37]. All these microorganisms are identified by the *T2 Biosystems* Bacteria Panel. The implementation of targeted antibiotic therapy alone has many implications. First of all, it increases the chances of achieving therapeutic success. Furthermore, *ESKAPE* pathogens have a high potential for acquiring resistance mechanisms [38]. Broad‐spectrum antibiotic therapy promotes the development of drug resistance [39]. Therefore, targeted antibiotic therapy will reduce the risk of resistance emergence, while also preserving gut microbiota, which is clinically significant. This role was demonstrated by Wienhold SM et al. in a murine model. They presented the correlation between lung injury, antibiotic therapy, and microbiota disruption [40]. Thus, there is no doubt that one of the priorities in the treatment of sepsis and septic shock should be to strive for the earliest possible identification of the pathogen and prompt implementation of targeted antibiotic therapy.

Sepsis remains one of the leading problems not only in intensive care unit patients but also in those with internal medicine and surgical profiles. Fast identification of high‐risk patients and accurate diagnosis lie at the heart of all dilemmas. Despite the necessity for immediate antibiotic therapy in cases of septic shock [41, 42, 43], there are literature reports indicating that there is no strong correlation between patient survival and the timing of antibiotic therapy initiation within the first hours after sepsis diagnosis [44]. However, these reports should not be interpreted as contradictory, but rather as complementary. The pathophysiology of sepsis and septic shock still raises many questions and requires further research. With the development of modern microbiological diagnostic techniques, it is evident that approaches to pathogen identification and antibiotic therapy will undergo changes. What remains unchanged is that the treatment of patients with sepsis and septic shock always requires a holistic and personalized approach. Certainly, there are many clinical situations where delaying antibiotic therapy for a few hours to target it toward the pathogen is highly justified and clinically feasible [45, 46].

## 5 Conclusions

Currently, in septic shock, according to the Surviving Sepsis Campaign guidelines, we should aim to administer antibiotics as soon as possible, ideally within the first hour of diagnosing septic shock. However, emerging studies suggest that this strategy may need to be re‐evaluated or at least refined [44].

Undoubtedly, it is essential to aim for the fastest possible stabilization of the patient's condition (e.g., fluid therapy with balanced crystalloids, administration of vasopressor drugs, etc.), as well as the prompt initiation of antimicrobial therapy. However, if the patient's condition is quickly stabilized with these interventions, it may be worth waiting for the T2Dx result to guide the antibiotic therapy. This is also linked to the fact that if sepsis is excluded as the cause of the patient's deterioration, it will prompt us to investigate other potential circumstances, which could expedite the diagnosis and implementation of appropriate treatment. Unfortunately, the symptoms accompanying sepsis can also occur in other diseases, which should always be considered when treating critically ill patients.

A single analysis of parameters such as CRP, WBC, #neutr, #lymph, and NLR is not a good indicator that could be used as an additional tool facilitating patient qualification for T2Dx testing. The most sensitive parameter distinguishing between patients with a positive T2Dx result and those with a negative result is the measurement of IL‐6 and PCT. Moreover, both IL‐6 and PCT exhibit significant dynamics in parameter changes over a short period (< 24 h), which can effectively facilitate the identification of patients who would benefit from T2Dx testing. The high dynamics of IL‐6 and PCT are associated with a greater likelihood of obtaining a positive T2Dx result. Proper patient qualification for T2Dx testing can significantly contribute to reducing the time to initiate targeted antibiotic therapy and may impact reducing mortality and improving long‐term treatment outcomes. Currently, there is still limited sufficient research available in the literature presenting experiences with the application of modern microbiological diagnostic methods using amplification and magnetic resonance technologies.

## Author Contributions

All authors made a significant contribution to the work reported, whether that is in the conception, study design, execution, acquisition of data, analysis, and interpretation, or in all these areas; took part in drafting, revising, or critically reviewing the article; gave final approval of the version to be published; have agreed on the journal to which the article has been submitted; and agree to be accountable for all aspects of the work.

## Ethics Statement

This study was conducted as part of standard clinical practice and as such does not require informed consent. However, the results were retrospectively collected from the patient database. We have received approval from the bioethics committee.

## Conflicts of Interest

The authors declare no conflicts of interest.

## Data Availability

The data that support the findings of this study are available on request from the corresponding author. The data are not publicly available due to privacy or ethical restrictions.
